# Actives from MMV Open Access Boxes? A suggested way forward

**DOI:** 10.1371/journal.ppat.1009384

**Published:** 2021-04-22

**Authors:** Kirandeep Samby, Paul A. Willis, Jeremy N. Burrows, Benoît Laleu, Peter J. H. Webborn

**Affiliations:** 1 Medicines for Malaria Venture, Geneva, Switzerland; 2 PJHW Consultancy Ltd, Marton, United Kingdom; Joan and Sanford I Weill Medical College of Cornell University, UNITED STATES

## Abstract

It is estimated that more than 1 billion people across the world are affected by a neglected tropical disease (NTD) that requires medical intervention. These diseases tend to afflict people in areas with high rates of poverty and cost economies billions of dollars every year. Collaborative drug discovery efforts are required to reduce the burden of these diseases in endemic regions. The release of “Open Access Boxes” is an initiative launched by Medicines for Malaria Venture (MMV) in collaboration with its partners to catalyze new drug discovery in neglected diseases. These boxes are mainly requested by biology researchers across the globe who may not otherwise have access to compounds to screen nor knowledge of the workflow that needs to be followed after identification of actives from their screening campaigns. Here, we present guidelines on how to move such actives beyond the hit identification stage, to help in capacity strengthening and enable a greater impact of the initiative.

## Introduction

More than 1 billion people globally are affected by neglected tropical diseases (NTDs), which leads to an economic loss of billions of dollars to developing economies [1–3], beyond the human tragedy. Current trends show that there are limited treatment options for many of these diseases, partly due to the emergence of resistant pathogens [4,5] but principally due to a lack of research and development (R&D) investment in drugs for diseases with little commercial value [3,6]. In addition, new pathogens can “jump” from animals to humans [7,8] creating an ever-present threat, as dramatically illustrated recently by the Coronavirus Disease 2019 (COVID-19) pandemic. These NTDs pose major public health risks, and effective drugs are needed to treat and control them [9]. The discovery of such new medicines for NTDs requires alternative drug discovery and development funding and implementation mechanisms beyond those driven solely by the pharmaceutical industry. Nevertheless, the robust and efficient drug discovery processes and quality control criteria that have been developed from years of work in pharmaceutical industry on diseases that primarily affect high income countries must still be applied. Given the restricted resources, these processes aid decision-making at every step of the drug discovery process and help prioritize the most interesting options to deliver safe, efficacious, and affordable NTD drugs.

There has been an increase in investment in the areas of NTDs and antimicrobial resistance, mostly by efforts from nonprofit foundations like the Bill & Melinda Gates Foundation, the Global Health Innovation Technology Fund, the Wellcome Trust, the RIGHT Fund, and various government agencies [10,11]. This has led to an increased focus on understanding the biology and pathogenesis and the development of phenotypic high-throughput assays to allow the screening of compound collections to generate hits. New target identification tools for establishing the mechanism of action of hits [12–17] identified through phenotypic screening have also helped fuel drug discovery efforts. Given the limited market incentives for R&D in infectious diseases in general, product development partnerships (PDPs) like Medicines for Malaria Venture (MMV), Drugs for Neglected Diseases *initiative* (DND*i*), and Global Alliance for Tuberculosis (TB Alliance) are partnering with pharmaceutical companies and academic groups to develop new drug discovery programs and screening platforms which have the potential to deliver new or more effective drugs [18,19]. In order to catalyze research in the area of infectious diseases and engage groups working on establishing novel screening systems, MMV provides open access boxes containing physical samples of up to 400 compounds to researchers all over the world, free of cost [20]. Since 2013, it has launched 4 open access boxes: the Malaria Box (2013 to 2015; [21,22]), the Pathogen Box (2016 to 2020; [23]), the Pandemic Response Box (2019 to present; [24]) in collaboration with DND*i*, and the COVID Box (2020 to present; mmv.org). The availability of these compound sets for screening not only enables scientists to identify new starting points for drug discovery programs or chemical biology tools but also helps in capacity building of groups in low- and middle-income countries.

The process of identifying drug candidates with a quality suitable for further clinical development is complex and lengthy with the optimization phase taking up to 2 years in hit to lead (HTL) and up to 3 years in lead optimization (LO) phases depending upon resources available. Evaluating a series against well-defined criteria can help in the identification of good quality preclinical candidates which can then be progressed further. Here, generic criteria have been proposed for validating hits emerging from screening of open access boxes which will help research groups to decide on the feasibility and attractiveness of progressing actives from a screening campaign to an HTL program [25].

### Hit identification

**Fig 1:**
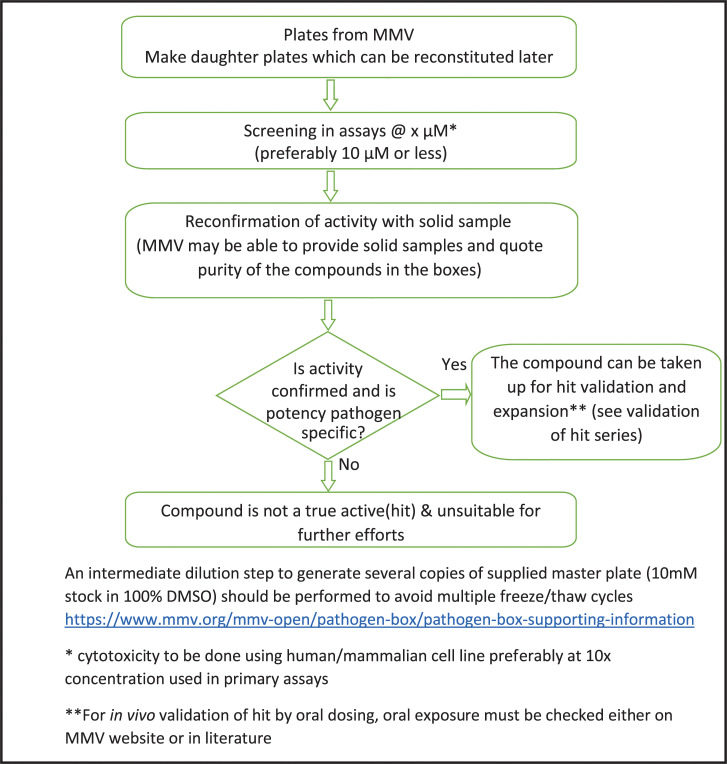
Suggested screening cascade of compounds from MMV Open Boxes. MMV, Medicines for Malaria Venture.

The first step to develop a new small molecule drug involves identifying the chemical starting point for subsequent optimization. Typically, this start point comes from the screening of compound libraries in validated assays (i.e., those where outcomes are relevant for the disease they address) to identify “actives” which have a suitable in vitro response in primary assays that are either target based or phenotypic. The assays should not only be biologically relevant but also robust and reproducible with Z’ value greater than 0.5. In these assays, a sigmoidal concentration target/growth inhibition curves reaching a maximal 100% potency can be generated; from this, an IC_50_ is calculated, being the concentration that achieves 50% inhibition. This in vitro potency needs to be critiqued and confirmed at the level of the compound and in terms of the biology: First, sufficient replicates are needed, ideally with a new solid compound sample with known high purity (>90%) to confirm the data and compound integrity. Second, it is important in such assessments to ensure that 100% inhibition is achieved at some concentration and that the concentration response is pharmacologically achievable. For infectious diseases, where actives are identified from target-based screens, it is recommended to confirm activity in cell-based screens predictive of the disease [26–30] along with an assessment of selectivity in a biochemical counter assay (for example, a homologous mammalian target, especially human). Cytotoxicity evaluation using a human or other mammalian cell line helps to confirm the specificity and that the compound is a true active (hit) (Fig 1). Cytotoxicity data on compounds from the Pathogen Box or Pandemic Response Box can be accessed at mmv.org/mmv-open/. Usually, a >10-fold selectivity window for cytotoxicity for the mammalian cell line is preferred for infectious diseases at the hit stage, but this criterion may be relaxed in the case of diseases where the hit rate is low and/or there are fewer compound inhibitor options [31].

On identifying a potent and selective active (hit) in phenotypic screening assays, researchers often plan immediately for in vivo validation with the initial compound itself. It is rare for such an initial hit to have sufficient plasma free drug concentrations after oral administration in relation to their pathogen potency to justify this due to high clearance and/or poor absorption. Thus, it is important for groups to first consider the rodent in vivo pharmacokinetic (PK) data for such a compound. In the case of the MMV Open Boxes, such information may already be available on the MMV website or in the literature. At that point, the most promising actives and formulations can be selected, setting doses and dose frequencies, to progress to in vivo disease models [32]. If the hits are very potent but have poor oral bioavailability, it is sometimes possible to increase exposure by administering the compound by alternate routes such as intravenous (i.v.)—which maximizes plasma exposure by avoiding incomplete intestinal absorption and first-pass metabolism, or intraperitoneal (i.p.) or subcutaneous (s.c.) which minimizes risks due to poor absorption. If in vivo exposure data are not available, then in vitro drug metabolism and solubility/permeability data may be used to predict whether a compound is likely to achieve suitable oral exposure, before testing in animals. In general, in vitro intrinsic clearance in mice or rat microsomes or hepatocytes that equate to a clearance of <50% liver blood flow (Table 1) and a solubility >10 μM are recommended before considering oral in vivo dosing. Literature evaluation of the broader pharmacology of the hit and the associated chemical series, based on chemical structures or substructures, provides information about the activity in other screens. Translation from in vitro to in vivo experiments is not always predictive or straightforward and can result in inefficacious compounds even though the compounds have cell-based activity and sufficient exposure. There is then a need to understand in vitro pharmacology parameters like potency in physiologically relevant settings, target engagement (if relevant), and appropriate in vivo models to name a few.

**Table 1:** In vitro data that would predict a hepatic extraction in the rat of 50%.

Species	System	Plasma binding (%free)	Cells per ml or mg/ml	Incubation time (min)	Turnover (%)	T 1/2 (min)	CL_int_ (μl/min/10^6^ cells) or (μl/min/mg)	Free AUC at 10 mg/kg p.o. (assuming 100% absorption)
Rat	Hepatocytes	20	0.5	60	37	90	15	580
Rat	Hepatocytes	10	0.5	60	50	60	23	290
Rat	Hepatocytes	5	0.5	60	75	30	46	145
Rat	Hepatocytes	1	0.5	60	98	10	139	30
Rat	Microsomes	20	0.5	45	32	80	17	580
Rat	Microsomes	10	0.5	45	25	55	25	290
Rat	Microsomes	5	0.5	45	55	40	35	145
Rat	Microsomes	1	0.5	45	80	20	69	30

The calculations are based on the well-stirred liver model, a blood:plasma ratio of 1, and microsomal binding commensurate with the plasma protein binding [47].

AUC, area under the curve.

### Validation of hits

Once individual compounds have been identified and confirmed to be active and specific, it is critical to establish whether the compounds have scope to be optimized further. As such, the broader chemical series around the hit needs to be explored (the “analogs”). This will then give confidence in the quality of scaffolds to take up for a formal drug discovery program, starting with an HTL phase. The ability of chemistry to easily generate derivatives (a series’ “tractability”) is an important factor as well here—this is often a major problem with compounds that have a natural origin [33]. The first way to expand around the hits is using “structure–activity relationship (SAR)” by catalog, wherein 20 to 30 similar compounds are purchased and screened. These similar compounds or “near neighbors” are selected by medicinal chemists to maximize the understanding of the impact of structural changes on potency and establish tractability. Naturally, a major constraint can be the commercial availability of such similar compounds. If near neighbors are not commercially available, the synthesis of a few designed analogs is recommended. Evaluation of these compounds in the primary screen helps to confirm that the identified hit is part of a broader series, to establish SAR tractability and shortlist scaffolds for further optimization. Preliminary SAR and cytotoxicity information will help teams to assess the strengths and weaknesses of the compound. For many of the compounds from the MMV Pathogen Box, small libraries of compounds related to the original structure were synthesized, and these can be provided to researchers to develop a preliminary SAR understanding. The objective is to understand the characteristics of the scaffolds in terms of disease specific (e.g., potency and selectivity), physicochemical, and PK properties (e.g., solubility or rate of metabolism). Developing an understanding of the chemical scope of the series is critical to determine whether a future drug discovery program is feasible. There may be multiple scaffolds available at this stage; in that case, evaluation of ligand efficiency or LE (how potent the compound is relative to its atom count or molecular weight) and lipophilic ligand efficiency or LLE (how potent a compound is relative to its lipophilicity) can help triage these hits [34]. These calculations are helpful because the higher the efficiency of binding (LE) or the lower its dependency on lipophilicity for potency (LLE), the greater the potential scope for optimization. If the initial hit contains a chemically reactive group or any other pan-assay interference compounds (PAINS) [35,36], it is important to understand the contribution of the reactive group to potency and other properties, since reactivity may be associated with toxicity through the irreversible formation of covalent bonds to endogenous proteins, with subsequent off-target effects [37]. Naturally, if the chemically reactive group can be removed or modified to reduce the risk of such pharmacological promiscuity while at the same time maintaining pathogen potency, then the series’ attractiveness can be dramatically improved. A novelty search based on an exact structure or scaffold with generic substitutions is important to ensure freedom to operate (an exact structure search can be done in databases like ChEMBL and PubChem, while generic search will require access to databases like SciFinder, Cortellis, or Clarivate Analytics). Although chemical novelty facilitates intellectual property claims, the new findings do not always need to be protected by patents. Patent protection will depend on the therapeutic area, stage of the project, and the population that is to be treated.

Hit validation of a series in infectious disease programs will always involve confirmation that compounds in the series are active on the pathogen in a relevant phenotypic assay, regardless of whether the hits were identified from a target-based screen. Compounds should be active in whole-cell screening assays and show sufficient selectivity in a human orthologue biochemical or phenotypic cytotoxicity counter assay. A suggested checklist for validating a hit series is detailed in Box 1.

**Box 1. General check points for validation of the hit series**

1. Establish the SAR tractability of the hit series and understand the chemical space for modification.
2. Novelty search based on exact compounds or scaffolds with generic substitutions
3. There should ideally be multiple points of modifications in the scaffold which provide options for further chemistry changes to improve properties such as absorption, distribution, metabolism, and excretion (ADME) and toxicity.
4. Hits should be devoid of highly reactive or unstable functional groups (groups responsible for promiscuous activity, PAINS).
5. Compounds should be amenable to chemical synthesis in relatively few synthetic transformations (e.g., <5–7 steps) with good yields. This helps minimize design–make–test cycle timelines. A low cost of goods is critical for neglected disease drugs, so chemical hits and series with multiple chiral centers, large number of synthetic steps, or requiring expensive starting materials may prove problematic unless these can be changed or simplified. A synthetic route which allows introduction of structural diversity in the final steps is beneficial as it simplifies the synthesis of final compounds from a common intermediate that can be prepared in a reasonable quantity.
6. For hits identified through target-based approaches, a preliminary understanding of the cell-based activity (whole-cell pathogen data) is required. The selectivity profile (cytotoxicity and selectivity with respect to human orthologues in case of target-based approach) also is needed. Literature searches may suggest potential off-target activities for evaluation.
7. Activity against drug resistant and sensitive strains, where available, should also be monitored.
8. An evaluation of the overall strengths and weaknesses of a hit series to aid prioritization and define the goals of the optimization program

Hit series should also be checked for their alignment with the disease target product profiles (TPP) as early as possible, even though a detailed biological and PK profile will be generated as the project progresses. For example, in the case of malaria, 2 TPPs and 6 target candidate profiles (TCPs) based on patient stratification and medical use case have been established [38–40]. The act of positioning a chemical series against a TCP helps to identify the relevance of pursuing a series based on its biological profile, as well as helping to map out the optimization considerations toward a disease-relevant and community-required drug candidate. These TPPs and TCPs for neglected diseases are generally defined by various PDPs in consultation with the larger scientific and medical community [31]. Detailed TPPs and TCPs for neglected diseases do not fall under the purview of this article. Criteria for validated hits will be different based on the targeted disease [41–43], but, as an example, the criteria for a validated hit for malaria are described in Table 2. Although these criteria are proposed, it is essential to regularly review and update TPPs and TCPs to align with any emerging data and patient needs.

**Table 2:** Criteria for validated hit series for malaria.

Compound property	Criteria
Purity and reconfirmation of activity	The activity of the identified hit should be confirmed using resynthesized/purchased compound with >90% purity and where the structure has been confirmed using analytical techniques SAR tractability established by synthesis or procurement of close analogs
Molecular weight	<500 Da
LogD pH 7.4 (measured)	<5
No. of H-bond donors	<5
No. of H-bond acceptors	<10
No. of stereogenic centers	<3 (to reduce the synthetic complexity and cost of goods at a later stage)
Solubility in PBS (pH7.4) (μM)	>10 μM
Genotoxicity risks based on structure, e.g., hidden anilines present?	No obvious risks based on in silico analysis
Novelty	Literature search on the exact compound and simplified core
Potency at in vitro biochemical target (Ki) if target-based approach is being followed	Ki or IC_50_<1,000 nM against *Pf* biochemical target if known and target assay available
Selectivity vs. relevant related human host target, if known (fold)	>10
Potency erythrocyte assay (EC_50_) − (minimum 7 strains including lab-derived and clinical mutants)	EC_50_<1,000nM (48 h, 72 h)
Cytotoxicity assay (EC_50_) − (minimum 2 cell lines) − selectivity index	>10-fold
Mechanism of action	Novel based on structure and phenotype, unless deliberately prioritized as a backup series against a known target
Additional activity	*Pv* hypnozoite (TCP3), *Pb*/*Pf* liver schizont (TCP4), and *Pf* male and female gamete formation activity (TCP5) to determine relevance for respective TCP (in vitro EC_50_<1,000 nM in relevant assays)
Profile in in vitro ADME assays to understand the clearance mechanism and rates of metabolism (mouse, rat, and and human).	

ADME, absorption, distribution, metabolism, and excretion; *Pb*, *Plasmodium berghei*; *Pf*, *Plasmodium falciparum*; *Pv*, *Plasmodium vivax*; SAR, structure–activity relationship; TCP, target compound profile.

Oral administration is generally the preferred dosing route for treating neglected diseases in nonhospital settings, so it is important to measure physicochemical properties such as solubility and the distribution constant which may indicate whether dissolution or permeation through the intestinal wall may be a limiting factor in the absorption of a high proportion of the administered dose. Clinical efficacy is usually associated with maintaining free drug levels above a minimum threshold for an extended period of time. How a compound is eliminated (and how fast) and how widely it distributes (the volume of distribution) are the factors that govern the concentration–time profile of free drug levels. In vitro assays in microsomes or hepatocytes to determine the intrinsic clearance (CL_int_) of a compound are convenient and commonly used assays to assess metabolic stability and can be used to drive drug design, if metabolism is the key elimination process of a chemical series. It is worth noting that although the emphasis is on free drug levels, modulating plasma protein binding itself is not an effective way to optimize [44]. Understanding the PK parameters that govern concentration–time profile and their dependence upon physicochemical properties helps to generate structure–property relationships that give confidence (or otherwise) that parameters can be optimized further, to deliver a clinically useful profile [45,46]. Researchers can also approach the PDPs who develop drugs against their pathogen of interest to obtain more information about the hits emerging out of open boxes and discuss the way forward.

During the HTL phase, the emphasis is on the improvement of compound properties including potency, selectivity, metabolic stability, permeability, solubility, cytochrome P 450 (CYP) inhibition, and safety parameters like hERG inhibition and genotoxicity (in case of structural alerts like presence of anilines). This helps to determine which series are likely to deliver a drug candidate and should progress to a full LO program. For infectious diseases, killing potential of hits (whether “cidal” or “static” and rate of killing) may help in series selection and suggest possible mechanisms of action for phenotypic hits. The HTL strategy adopted can be very different, based on the profile of the initial hit and should focus on addressing the specific issues present, highlighting the need for detailed screening to evaluate the particular strength and issues. Compounds which meet the target potency, selectivity, and in vitro absorption, distribution, metabolism, and excretion (ADME) parameters should next be assessed for i.v. and oral (p.o.) in vivo PK to help select compounds for proof of concept in animal models and to further understand the PK properties of the series. This may potentially highlight other issues for further optimization such as lack of oral absorption.

If the pathogen is located in a particular tissue, specific properties may be required to ensure high drug concentrations in that compartment like drugs needing to cross the blood–brain barrier have lower molecular weights, lipophilicity and hydrogen bond donors, and acceptors than other therapeutics [48]. Cryptosporidium primarily infects the epithelial layer of the small intestine so it has been proposed that new drugs with high gastrointestinal exposure and low systemic exposure would be optimal for achieving adequate efficacy and safety properties [42].

Discovery work does not stop at the lead identification stage, but the scaffolds have to be further improved during the LO stage. The objective in this stage is to retain favorable properties in the compounds while working on their deficiencies to identify compounds with sufficient potency, efficacy, exposure, and safety margins. In general, compounds need to be profiled in models of genotoxicity, safety pharmacology, PK/PD, and drug-induced metabolism before being taken forward for preclinical development studies which involves further characterization in pharmacology, toxicology, and developability studies [12,31,49].

## Conclusion and future directions

The process from hit identification to preclinical candidate selection for NTDs often takes a long time, depending on resources available, and rarely has shortcuts. There are few examples of drug repositioning, where compounds have progressed directly from phenotypic screening to patients because their human PK and clinical safety profile were well known, but in those cases, sufficient preclinical toxicity data and at times Phase I human data would be available to make such decisions [50]. The HTL process also involves significant intellectual input from scientists from various disciplines and experience, working together in a drug discovery project. The quality of the selected hits for further optimization often defines the path for project progression and is a key determinant for successful identification of leads. We have described a selection cascade to prioritize hits resulting from screening of diverse libraries. As an example, a proposed criterion for validated hits for malaria, which can facilitate decision-making by the project teams, is described. To aid decision-making, in vitro ADME and in vivo PK of the compounds from MMV box projects are available online (https://www.mmv.org/mmv-open). Scientists are encouraged to contact MMV (MMVopen@mmv.org) once hits have been identified from these open boxes so that they can be supported with drug discovery advice.
